# The Impact of Amniotic Fluid Interleukin-6, Interleukin-8, and Metalloproteinase-9 on Preterm Labor: A Narrative Review

**DOI:** 10.3390/biomedicines13010118

**Published:** 2025-01-07

**Authors:** Theodoros Karampitsakos, Despoina Mavrogianni, Nikolaos Machairiotis, Anastasios Potiris, Periklis Panagopoulos, Sofoklis Stavros, Panos Antsaklis, Peter Drakakis

**Affiliations:** 1 Third Department of Obstetrics and Gynecology, University General Hospital “ATTIKON”, Medical School, National and Kapodistrian University of Athens, 124 62 Athens, Greece; nikolaosmachairiotis@gmail.com (N.M.); apotiris@med.uoa.gr (A.P.); perpanag@med.uoa.gr (P.P.); sfstavrou@med.uoa.gr (S.S.); pdrakakis@med.uoa.gr (P.D.); 2 First Department of Obstetrics and Gynecology, Alexandra Hospital, Medical School, National and Kapodistrian University of Athens, 115 28 Athens, Greece; dmavrogianni@med.uoa.gr (D.M.); panosant@gmail.com (P.A.)

**Keywords:** interleukin-6 (IL-6), interleukin-8 (IL-8), metalloproteinase-9 (MMP-9), preterm labor, amniotic fluid

## Abstract

Background/objectives: Preterm labor is a leading cause of neonatal morbidity and mortality worldwide. Previous research has indicated that an inflammatory response or microbial invasion of the amniotic cavity is a pathological condition linked to preterm birth; hence, inflammatory markers such as metalloproteinase-9 (MMP-9), interleukin-6 (IL-6), and interleukin-8 (IL-8) have been utilized to predict preterm delivery. The identification of reliable biomarkers for early prediction is critical for improving maternal, fetal, and neonatal outcomes. Methods: To address this issue, a literature review has been conducted on PubMed/Medline and Scopus databases for articles investigating the possible correlation between IL6, IL8, and MMP9 and preterm labor. Results: Using a comprehensive search of the PubMed and Scopus databases, 12 studies were analyzed to identify the correlation between these biomarkers and preterm labor. Seven studies point the impact of increased IL-6 levels or polymorphisms of the gene and higher incidence of preterm labor. Two of the included studies identified the increased risk for preterm birth in elevated levels of IL-8 in amniotic fluid. Six studies highlight the increased incidence of preterm birth in women with polymorphisms of the MMP-9 gene. Conclusions: Elevated IL-6 levels and specific gene polymorphisms are strongly associated with preterm delivery risk, with IL-8 concentrations correlating with systemic inflammation and histologic chorioamnionitis. MMP-9 gene variations and protein levels showed significant predictive value for membrane rupture and labor onset. The findings emphasize integrating these biomarkers into diagnostic tools for routine prenatal care, enhancing early detection, risk stratification, and timely interventions to improve maternal and neonatal outcomes.

## 1. Introduction

Preterm labor, a significant cause of neonatal morbidity and mortality globally, remains a major challenge in obstetric care due to the lack of reliable predictive tools for early identification. While the role of inflammatory responses and microbial invasion in preterm birth has been well established, the translation of these findings into clinical practice has been limited. This is due to several gaps in current knowledge, including variability in biomarker expression, the influence of genetic polymorphisms, and the lack of standardized diagnostic thresholds. Maternal blood biomarkers are critical tools in understanding pregnancy outcomes, as they provide insight into the biological processes affecting both maternal and fetal health. These biomarkers, measurable parameters in blood samples, reflect the impact of various exposures or conditions during pregnancy and are linked to outcomes such as preterm birth, low birth weight, and infants that are small for their gestational age. Studies highlight the roles of inflammation-related markers and growth factor-/hormone-related markers in influencing these outcomes. By elucidating these associations, researchers aim to improve early diagnosis, guide preventive interventions, and better understand the underlying mechanisms affecting fetal development and maternal health [1,2]. Ιntra-amniotic inflammation due to microbial invasion of the amniotic cavity is a major cause of spontaneous preterm delivery. Proteins detected in amniotic fluid, mainly cytokines, are key regulators of parturition, and variations in their concentrations, independently of an infection at both term and preterm, have been well characterized. Initially it was proposed that regulatory activity of these proteins was mediated via different signaling pathways by direct interaction with cell-surface receptors. Actually, it is well established that such mediators are also associated with extracellular vesicles and are present both on the surface and within the lumen of vesicles [3].

Previous research has indicated that an inflammatory response or microbial invasion of the amniotic cavity is a pathological condition linked to preterm birth [4]. While the molecular mechanisms through which intrauterine inflammation induces preterm birth remain unclear, several cytokines, chemokines, and inflammatory mediators have been implicated [5]. However, no biomarker has consistently proven useful in predicting spontaneous preterm delivery in asymptomatic women, particularly during the second trimester [6]. A narrative review focusing on biomarkers such as interleukin-6 (IL-6), interleukin-8 (IL-8), and matrix metalloproteinase-9 (MMP-9) is warranted to bridge these gaps. These markers have demonstrated potential in identifying intra-amniotic inflammation and predicting preterm birth. However, their integration into clinical practice has been hindered by inconsistent data, the heterogeneity of studied populations, and the absence of robust, validated point-of-care tools [7]. Numerous studies have demonstrated that amniotic fluid (AF) IL-6 concentrations are more effective than AF white blood cell (WBC) counts, glucose levels, or Gram stains—and are comparable to proteomic markers—in detecting intra-amniotic infection and microbial invasion of the amniotic cavity (MIAC). Thus, AF IL-6 concentrations offer both diagnostic and prognostic value [8,9].

Elevated levels of IL-1β, IL-6, IL-8, and IL-10 in umbilical cord blood and early postnatal blood are associated with intraventricular hemorrhage and white matter lesions shortly after birth. Persistently elevated pro-inflammatory proteins during the first two weeks following preterm birth are also linked to an increased risk of cerebral palsy and impaired neurocognitive development in infancy and childhood [9]. Furthermore, specific preterm birth, such as intrauterine inflammation (histologic chorioamnionitis, HCA) and necrotizing enterocolitis, are associated with abnormal white matter observed on magnetic resonance imaging (MRI) [10]. On the other hand, Matrix metalloproteinases (MMPs) have a major role in the degradation process. These proteins are a family of extracellular proteolytic enzymes involved in different developmental and disease-related processes. The most frequently reported are MMP-1 and MMP-9, with the later reported to be induced during active labor and to play an important role in separation of the placenta from the uterus during delivery. Studies have shown high expression of MMP-1 and MMP-9 in different human fluids, including amniotic fluid, of women with PTB when compared to women with term delivery [11,12,13].

The aim of this review is to explore the role of specific biomarkers, particularly IL-6, IL-8, and MMP-9, in the prediction and management of preterm birth (PTB). This review aims to assess the diagnostic and prognostic value of these biomarkers in identifying women at risk of preterm delivery and to evaluate their potential integration into clinical practice for improving maternal and neonatal outcomes. Additionally, this review seeks to examine the underlying molecular mechanisms and genetic factors associated with PTB, with the goal of enhancing early detection and intervention strategies [4].

## 2. Literature Research

This narrative review was conducted using peer-reviewed primary research articles published up to March 2024 and focused on the impact of amniotic fluid IL6, IL8, and MMP9 on preterm birth. A thorough literature search was performed using the PubMed/Medline and Scopus databases, employing keywords such as “IL6”, “IL-6”, “Interleukin 6”, “IL8”, “IL-8”, “Interleukin 8”, “MMP9”, “MMP-9”, “Matrix metalloproteinase-9”, “amniotic fluid”, “preterm labor”, “preterm delivery”, and “preterm birth”. These terms were either used separately or in combination with the help of the Boolean administration (OR, AND). All articles with an English title and abstract were initially accepted. Case reports, editorials, and animal studies were excluded from this review. Additionally, the “snowball literature searching method” was applied to identify further relevant sources from the reference lists of selected articles.

**Figure 1:**
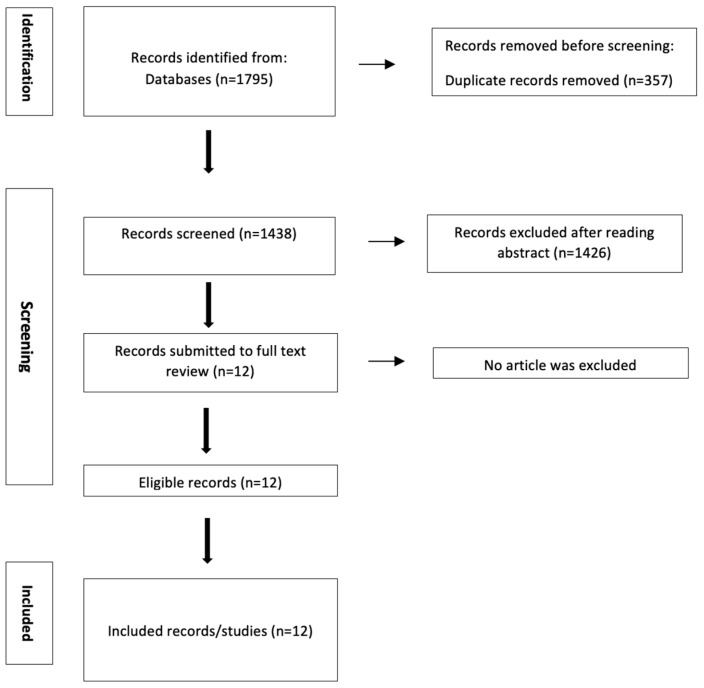
Schematic illustration of the study selection process.

In total, 1795 articles were retrieved via different databases and 1438 were assessed after duplicate exclusion. The initial assessment of titles and abstracts was performed by two independent reviewers, T.K. and D.M. Subsequently, a full-text assessment was performed. If a study was selected only by one reviewer, the decision was taken by a third reviewer (N.M.). Ultimately, twelve records were selected for full text assessment and included in this literature review. Figure 1 presents the study selection process. A formal risk of bias and a quality assessment were not performed due to the narrative nature of this review.

## 3. Results

Chaemsaithong P et al. evaluated the efficacy of a point-of-care (POC) test for measuring amniotic fluid (AF) interleukin-6 (IL-6) concentrations to identify intra-amniotic inflammation and predict spontaneous preterm delivery in women with preterm labor and intact membranes. The research included 136 women with singleton pregnancies who underwent amniocentesis. The study compared the diagnostic performance of the POC test with the conventional enzyme-linked immunosorbent assay (ELISA). Finaly, in Chaemsaithong P et al.’s study, the POC IL-6 test showed high sensitivity (93%) and specificity (91%) for detecting intra-amniotic inflammation, with a rapid turnaround time, making it a valuable tool for predicting imminent preterm delivery and enabling timely clinical interventions [9].

Furthermore, Wilfred Wu et al. investigate the association between the IL6 gene polymorphism (specifically SNP rs1800795) and the risk of preterm birth (PTB). The study conducted a meta-analysis of existing research, stratifying the analysis by population to account for differences in genetic background and increase statistical power. The results indicate that the CC genotype of rs1800795 is protective against PTB in women of European descent, with an odds ratio (OR) of 0.68. However, this protective effect was not observed in women from other populations or in analyses of fetal genotypes. The study highlights the importance of considering population structure in genetic studies of PTB [14].

Also, Teresa Cobo et al. focused on developing a rapid and effective diagnostic method for identifying intra-amniotic inflammation in women experiencing preterm labor (PTL) with intact membranes. The researchers aimed to validate the use of an automated immunoassay to measure amniotic fluid (AF) interleukin-6 (IL-6) concentrations, which was previously conducted using the ELISA method. In this study, 100 women with PTL under 34 weeks who underwent amniocentesis were included. The addition of IL-6 in a multivariable predictive model improves the identification of women at high risk of PTL within 7 days, potentially optimizing antenatal care and management. The article focuses on validating an automated immunoassay method to measure amniotic fluid interleukin-6 (AF IL-6) in women with preterm labor (PTL). This predictive model, which also considers gestational age, cervical length, and glucose levels, could be instrumental in identifying women at high risk for early delivery, potentially optimizing clinical management and improving perinatal outcomes. The study underscores the importance of integrating rapid and reliable diagnostic tools in clinical practice to manage preterm labor effectively [15].

Xia Qiu et al. investigated the diagnostic value of interleukin-6 (IL-6) for detecting neonatal sepsis in cases of premature rupture of the membranes (PROM). This meta-analysis includes data from multiple studies to assess the accuracy of IL-6 as a biomarker. The findings reveal that IL-6 has high sensitivity (0.85) and specificity (0.88) for early diagnosis, with a diagnostic odds ratio (DOR) of 79.26 and an area under the summary receiver operating characteristic (SROC) curve of 0.9473. These results indicate that IL-6 is a reliable marker for the early identification of neonatal sepsis in the context of PROM, suggesting its potential for improving clinical outcomes by enabling timely intervention [16].

The article investigates the role of IL-6 and its interaction with matrix metalloproteinases (MMPs) in preterm birth (PTB). The study explored the interactions of IL-6 with MMP-1, MMP-8, and MMP-9 and found that certain polymorphisms in these genes, particularly IL-6 and MMP-9, were significantly associated with increased risk of PTB. IL-6 polymorphisms, especially the presence of the G allele, showed a strong association with elevated MMP-9 levels and PTB. The research suggests that the combination of IL-6 and MMP-9 gene polymorphisms can be a potential marker for predicting the risk of PTB. Overall, IL-6 affects preterm labor by promoting inflammatory pathways and interacting with MMPs to induce degradation in the extracellular matrix, leading to premature rupture of membranes and labor onset [17].

Another study examined the relationship between midtrimester amniotic fluid concentrations of IL-6 and interferon-gamma-inducible protein-10 (IP-10) with spontaneous preterm delivery. The main findings are the relevance of preterm delivery. Overall prevalence of preterm delivery was 8.3%. Early spontaneous preterm delivery (≤32 weeks) occurred in 1.5% (n = 12) and late spontaneous preterm delivery (>32 weeks) occurred in 4.5% (n = 36). The association with preterm delivery showed that an elevated IL-6 concentration (≥1740 pg/mL) was associated with early spontaneous preterm delivery with an odds ratio of 9.5 (95% CI 2.9–31.1). Furthermore, no detectable microorganisms were found in the amniotic fluid using cultivation techniques [6].

IL-8 significantly affects preterm labor through its role in systemic inflammation and its association with histologic chorioamnionitis (HCA), a leading cause of preterm birth. The study found that elevated IL-8 levels in the first week of postnatal life are associated with dysmaturation of white matter at term-equivalent age. This dysmaturation can result in impaired cognitive development and psychiatric disorders in preterm infants. The link between IL-8 and altered white matter development underscores the importance of this cytokine in the pathophysiology of preterm brain injury and its potential as a target for therapeutic intervention [10].

The study by Hong et al. focuses on identifying proteins in the amniotic fluid (AF) that are linked to the rupture of membranes (ROM) in women experiencing threatened preterm labor (PTL). Using an antibody microarray, researchers conducted a retrospective cohort study involving 183 pregnant women with PTL who underwent amniocentesis. The study identified 17 proteins with significant differences between women who experienced ROM within 7 days and those who did not. Key findings included higher levels of EN-RAGE, Fas, IL-8, IP-10, MMP-8, and MMP-9 and lower levels of IGFBP-3 in women with ROM. These proteins are associated with inflammation, angiogenesis, matrix degradation, and apoptosis. The study’s results provide insights into the molecular mechanisms underlying ROM without active labor in threatened PTL, highlighting potential biomarkers for predicting and managing this condition [18].

José Duran-Chávez et al. investigated the association of matrix metalloproteinases (MMP-2 and MMP-9) levels in plasma and vaginal secretions with the risk of preterm birth (PB). They examined the association between levels of matrix metalloproteinases (MMP-2 and MMP-9) in plasma and vaginal secretions with preterm birth. It included 129 cases of preterm birth and 258 term controls. Elevated plasma levels of MMP-9 were significantly associated with preterm birth (odds ratio: 3.26), while vaginal MMP levels were not predictive. Plasma MMP-2 levels were also associated with increased risk of preterm birth, although to a lesser extent (odds ratio: 1.75). The research found that elevated plasma MMP-9 levels were significantly associated with an increased risk of preterm birth, nearly tripling the risk after adjusting for factors such as maternal age, parity, smoking, previous preterm birth, bacterial vaginosis, urinary tract infection, and cervical length. Conversely, plasma MMP-2 levels showed a moderate increase in PB risk, while no significant association was observed for MMP levels in vaginal secretions. The study underscores the potential of plasma MMP-9 as a predictive biomarker for preterm birth, highlighting the need for further research to validate these findings for clinical application [19].

The article evaluates the relationship between elevated maternal serum concentrations of IL-6, CRP, and MMP-9 and the risk of preterm birth (PTB) before 32 weeks, as well as associated neonatal morbidities. Elevated maternal serum concentrations of IL-6 and CRP were significantly associated with an increased risk of PTB before 32 weeks (IL-6: OR 4.60, CI 1.86–10.68; CRP: OR 4.07, CI 1.63–9.50). High levels of IL-6 and CRP were also linked to a higher risk of neonatal IVH, but were not significantly associated with RDS, CLD, NEC, or sepsis after adjusting for gestational age at delivery. The research was conducted on serum samples from 475 women at risk for PTB who were part of a multicenter randomized controlled trial. The findings indicate that high levels of IL-6 and CRP, but not MMP-9, are significantly associated with an increased risk of PTB < 32 weeks and neonatal intraventricular hemorrhage (IVH). However, these associations were not significant for respiratory distress syndrome (RDS), chronic lung disease (CLD), necrotizing enterocolitis (NEC), or sepsis after adjusting for gestational age. The study concludes that elevated maternal IL-6 and CRP levels are potential biomarkers for predicting early PTB and IVH, highlighting their importance in risk assessment and early intervention strategies (15). The study explored gene-gene interactions of IL-6 with MMP-1, MMP-8, and MMP-9 and found that certain polymorphisms in these genes, particularly IL-6 and MMP-9, were significantly associated with increased risk of PTB. IL-6 polymorphisms, especially the presence of the G allele, showed a strong association with elevated MMP-9 levels and PTB. The research suggests that the combination of IL-6 and MMP-9 gene polymorphisms can be a potential marker for predicting the risk of PTB. Overall, IL-6 affects preterm labor by promoting inflammatory pathways and interacting with MMPs to induce degradation in the extracellular matrix, leading to premature rupture of membranes and labor onset [20].

Additionally, in Moeini N et al.’s study, the role of vitamins B9 and B12 in the methylation and expression of the MMP-9 gene in women experiencing preterm birth was investigated. The study involved 100 pregnant women divided into two groups: 50 women who delivered preterm, and 50 who delivered at term. The researchers measured the levels of MMP-9 RNA and protein in placental tissues, as well as the methylation status of the MMP-9 promoter region. They found that women who delivered preterm had significantly higher levels of MMP-9 RNA and protein, along with lower methylation levels of the MMP-9 promoter, compared to those who delivered at term. Additionally, a significant negative correlation was observed between serum levels of vitamins B9 and B12 and the hypomethylation of the MMP-9 promoter. The study concludes that deficiencies in vitamins B9 and B12 may contribute to the hypomethylation and overexpression of the MMP-9 gene, which in turn may lead to preterm birth. In Moeini N et al.’s study, women delivering preterm had significantly higher MMP-9 RNA and protein levels and lower methylation levels of the MMP-9 promoter compared to those delivering at term. A significant negative correlation was observed between the serum levels of B9 and B12 vitamins and the hypomethylation of the MMP-9 promoter (B9: r = −0.48, *p* < 0.01; B12: r = −0.63, *p* < 0.001), indicating that deficiencies in these vitamins may contribute to PTB by affecting gene methylation and expression [13].

Yang Y. et al. examined the relationship between single nucleotide polymorphisms (SNPs) in the MMP2 (-735C>T) and MMP9 (-1562C>T) genes and the risk of recurrent spontaneous abortion (RSA). Through a meta-analysis of studies published before 17 April 2020, involving 528 RSA cases and 515 controls for MMP2 and 669 RSA cases and 661 controls for MMP9, the research aimed to determine whether these genetic variations are linked to RSA risk. The results reveal significant associations for both polymorphisms under various genetic models, suggesting that the MMP2-735T and MMP9-1562T alleles are associated with an increased risk of RSA. These findings underscore the potential role of these SNPs in RSA, although further studies with larger sample sizes are needed to confirm these associations and improve understanding of their mechanisms in the pathogenesis of RSA [21]. Table 1 summarizes the associations between IL-6, IL-8, and MMP-9 and the risk for preterm labor.

## 4. Discussion

A point-of-care (POC) test for amniotic fluid IL-6 can effectively detect intra-amniotic inflammation, as confirmed using ELISA, in women experiencing preterm labor with intact membranes. Additionally, this test shows comparable performance in identifying women who subsequently deliver spontaneously before reaching full term. Further research is necessary to determine whether the results of the POC AF IL-6 test can guide treatment decisions that improve pregnancy outcomes in these patients [9].

Wu W et al. conducted a study to investigate the association between preterm birth (PTB) and a polymorphism in the IL6 promoter region, specifically SNP rs1800795. The analysis was stratified by population subgroup and focused on early PTB. The findings reveal that the derived CC genotype is protective against PTB in women of European ancestry. However, no significant associations were observed in non-European samples, where the frequency of the CC genotype is lower [14].

Rapid diagnostic tests for intra-amniotic inflammation, such as the POC IL-6 test, offer promising advancements in the timely identification and management of at-risk pregnancies. Cobo, T et al. validated the use of an automated immunoassay for measuring amniotic fluid (AF) interleukin-6 (IL-6) levels in women with preterm labor (PTL) compared to the traditional ELISA method. It included 100 women, 38 of whom delivered within 7 days of admission. Both methods showed good agreement in measuring AF IL-6 concentrations (intraclass correlation coefficient: 0.937). The diagnostic performance of AF IL-6 in predicting spontaneous delivery within 7 days was robust, with an area under the receiver operating characteristic curve (AUC) of 0.894. The sensitivity was 97%, and the specificity was 74% [15].

Qiu, X et al. highlight the effectiveness of interleukin-6 (IL-6) as an early diagnostic marker for neonatal sepsis in cases of premature rupture of the membranes (PROM). Traditional methods like blood cultures are time-consuming and have low sensitivity, necessitating faster and more reliable biomarkers. IL-6 shows high sensitivity (0.87) and specificity (0.88), with a strong diagnostic odds ratio (DOR) of 79.26 and an area under the summary receiver operating characteristic (SROC) curve of 0.9473, indicating excellent diagnostic accuracy. Compared to C-reactive protein (CRP) and procalcitonin (PCT), IL-6 performs better for early sepsis diagnosis. CRP and PCT have lower sensitivity and specificity and are less reliable for early detection. IL-6, however, can be rapidly detected in small blood volumes, making it more practical for early diagnosis. The study found that IL-6 levels in neonatal peripheral blood were more accurate than in umbilical cord blood [16].

Pandey, M et al., by applying the Restriction Fragment length polymorphism (RFLP) and Generalized Multifactor Dimensionality Reduction (GMDR) methods, revealed that increased expression of MMP-9 was detected in those mothers carrying the IL-6 G allele, showing a synergistic effect in the increase in MMP-9 levels [17].

Therefore, prevalence and biomarkers in preterm delivery highlight the heterogeneity of intra-amniotic inflammation and its associations with preterm delivery. The differentiation between early and late preterm deliveries based on IL-6 and IP-10 concentrations can aid in risk stratification and management of pregnant women undergoing midtrimester amniocentesis. The absence of detectable microorganisms in most amniotic fluid samples suggests that non-infectious causes of inflammation, such as damage-associated molecular patterns (DAMPs) and sterile inflammatory responses, may play a role [6].

In Sullivan G et al.’s study, they propose that elevated IL-8 levels in umbilical cord blood were predictive of histologic chorioamnionitis, a leading cause of preterm birth. Additionally, high IL-8 levels in the first week of postnatal life were associated with white matter dysmaturation at term-equivalent age [10].

According to Hong et al., after using microarrays, they revealed that EN-RAGE, Fas, IGFBP-3, IL-8, and MMP-9 in the AF were independently associated with subsequent ROM in women with threatened PTL. AF IL-8 levels were independently associated with subsequent PPROM that occurred more than 7 days after a threatened PTL episode. Consequently, these data provide novel insights into the molecular mechanisms underlying membrane rupture without active labor in threatened PTL and propose a new therapeutic approach [18,19].

Yang Y. et al. revealed significant associations between the polymorphisms in MMP2 and MMP9 genes and the risk of RSA, suggesting that these genetic variations contribute to adverse pregnancy outcomes and potentially to PTB [21].

Sorokin Y. et al. proposed that elevated concentrations of IL-6, but not MMP-9, are associated with PTB < 32 weeks of gestation and subsequent development of intraventricular hemorrhage (IVH) in neonates. This study provided new information about IL-6 in early preterm delivery (<32 weeks), as even after adjustments for GA at delivery, elevated IL-6 confers an additional risk for IVH [20].

IL-6 and IL-8 are pivotal cytokines involved in the inflammatory pathways associated with preterm labor (PTL), a leading cause of neonatal morbidity and mortality. Inflammation is a well-established trigger for PTL, and both IL-6 and IL-8 have distinct but interconnected roles in this complex process. Experimental studies suggest that targeting IL-6 signaling could delay preterm delivery and improve maternal and fetal outcomes [22]. IL-8, primarily recognized for its role in recruiting and activating neutrophils, also contributes to the inflammatory milieu of PTL. Elevated maternal IL-8 levels correlate with adverse maternal health markers, including higher BMI, unhealthy diets, and gestational diabetes, although its direct association with spontaneous preterm birth has not consistently reached statistical significance. This suggests that IL-8 may influence PTL in specific contexts or critical time windows not fully captured in current studies. Despite these uncertainties, IL-8 remains a promising candidate as part of a biomarker panel to monitor inflammatory status and pregnancy outcomes [23].

The reviewed studies collectively highlight the multifactorial nature of PTB, involving inflammatory pathways, genetic predispositions, and nutritional deficiencies. Elevated IL-6 and CRP levels are robust predictors of PTB and associated neonatal complications, emphasizing the importance of these markers in early risk assessment and management. Genetic polymorphisms in MMP genes further contribute to the susceptibility to PTB, suggesting the need for genetic screening in high-risk populations. The impact of B9 and B12 vitamin deficiencies on gene methylation and expression underscores the significance of adequate maternal nutrition in preventing PTB [13,24].

## 5. Conclusions

Inflammatory markers, genetic polymorphisms, and micronutrient deficiencies play critical roles in the pathogenesis of preterm birth. Elevated levels of IL-6 and CRP and genetic variations in MMP genes are significant risk factors for PTB. Integrating these biomarkers and rapid diagnostic tools into routine prenatal care can improve early detection and intervention strategies, ultimately enhancing maternal and neonatal outcomes. These studies collectively underscore the critical role of inflammatory markers and advanced diagnostic tools in predicting and managing preterm births. The integration of these biomarkers into predictive models offers promising avenues for improving perinatal care and outcomes. Further research should focus on refining these models and exploring the underlying mechanisms of inflammation in preterm labor to develop targeted interventions and therapies.

## Figures and Tables

**Table 1:** Summary of associations between IL-6, IL-8, and MMP-9 and the risk for preterm labor.

Marker	Biological Source	Observed Level	Associated Risk	Study Findings/Notes
IL-6	Plasma	Elevated	Significant predictor for PTL/PTB	High maternal serum IL-6 associated with increased PTB risk (OR 4.60)
IL-6	Amniotic fluid	Elevated (>1740 pg/mL)	A 9.5-fold increase in early PTL risk	Strongly predictive of early spontaneous PTL
IL-8	Amniotic fluid	Elevated	Predictive if histologic chorioamnionitis	Linked to systemic inflammation and white matter dysmaturation in neonates
IL-8	Umbilical cord blood	Elevated	Predictive of brain dysmaturation in neonates	Elevated postnatally, associated with altered white matter development
MMP-9	Plasma	Elevated	Triples PTB risk (OR 3.26)	Plasma levels strongly associated with PTB
MMP-9	Vaginal secretions	Elevated	No significant association	Vaginal levels not predictive

## Data Availability

No new data were created or analyzed in this study.
